# Pseudoaneurysm of Left Ventricle following Staphylococcal Pericarditis in a Child

**Published:** 2015-09-01

**Authors:** Kamaldeep Arora, Rashmi Ranjan Das, Rohit Tandon, Kajal Goyal, Shasanka Shekhar Panda

**Affiliations:** 1 Department of Pediatrics, Dayanand Medical College and Hospital, Ludhiana - 141001, India; 2 Department of Pediatrics, All India Institute of Medical Sciences, Bhubaneswar - 751019, India; 3 Department of Cardiology, Dayanand Medical College and Hospital, Ludhiana - 141001, India; 4 Department of Paediatric Surgery, All India Institute of Medical Sciences, Bhubaneswar - 751019, India

**Keywords:** Staphylokinase, Pseudo-aneurysm, Pediatric, Echocardiography

## Abstract

Formation of pseudo-aneurysm of the left ventricle is a rare entity particularly in the pediatric age group. We report a case of a pseudo-aneurysm of the left ventricle in a 6-year-old boy who initially presented to us with staphylococcus aureus septicemia. The left ventricular pseudo-aneurysm was surgically resected and the boy was discharged in a healthy condition.

## INTRODUCTION

Pseudo-aneurysm in the left ventricle, a rare entity, may be an outcome of infective endocarditis, trauma or cardiac surgery. The occurrence of left ventricular pseudo-aneurysm as sequelae to staphylococcal septicemia with pericardial involvement is even rare. The first case was reported by Sadan et al in 1981.[1-8] In this report, we describe the diagnosis and management of a case of pseudo-aneurysm of the left ventricle.

## CASE REPORT

A 6 years old boy was admitted with a diagnosis of septicemic shock. There was history of trauma over the right ankle few days back with development of an abscess. The boy was taking treatment for the same from a quack. At admission, he was in altered sensorium with decompensated shock. Fluid boluses were given followed by ionotropic support, and he was put on mechanical ventilation. Intravenous antibiotics (ceftriaxone and vancomycin) were given after sending the blood culture. Examination revealed hepatomegaly (liver 4 cm below right costal margin with span of 14 cm), and bilateral crackles over the lung fields with slightly muffled heart sounds without any murmur. His hemoglobin was 11.2 g/dl, total leucocyte count 23,000/cumm (85% neutrophils), and platelet count 92,000/cumm. Acute phase reactants (C reactive protein = 84 mg/dl, ESR = 94 mm/1st hour) were elevated, but his renal and liver function tests including the urine examinations were normal. Chest x-ray showed cardiomegaly with blunted cardio-phrenic angles. Echocardiography done showed mild to moderate pericardial effusion with dense strands without any vegetations. The ankle abscess was drained, and the culture from the pus grew methicillin-resistant staphylococcus aureus (MRSA) as was the blood culture. Gradually, he responded to the above treatment and got discharged after 3 weeks of hospitalization with the advice to follow-up.

On the 2nd follow up visit (8 weeks after admission), he complained of palpitations and dyspnoea that was gradual in onset, but progressive in nature. On examination, he had tachycardia, audible S3, early to mid systolic murmur at lower sternal region, and bilateral basal crepitations. The severity was classified as of NYHA class III. An urgent echocardiogram revealed a large (34x22 mm) inferior septal pseudo-aneurysm with narrow neck (5 mm) projecting posteriorly, and communicating with the left ventricular cavity in close proximity to anterior mitral valve leaflet (Fig. 1-3). He was hospitalized, put on decongestive measures, and immediate surgery was planned. During surgery, a large aneurysmal sac (5.1x4.7x4.1cm) communicating with the left ventricle and compressing the coronary sinus, right atrium, and inferior vena cava junction was found. The sac was resected. On inspection, the pericardium was thick and inflamed, and there was right para-tracheal, pre- and sub-carinal lymphadenopathy. The post-operative course was uneventful and the boy got discharged in a healthy condition. He is currently in follow-up for the last 2 years and is asymptomatic.

**Figure:**
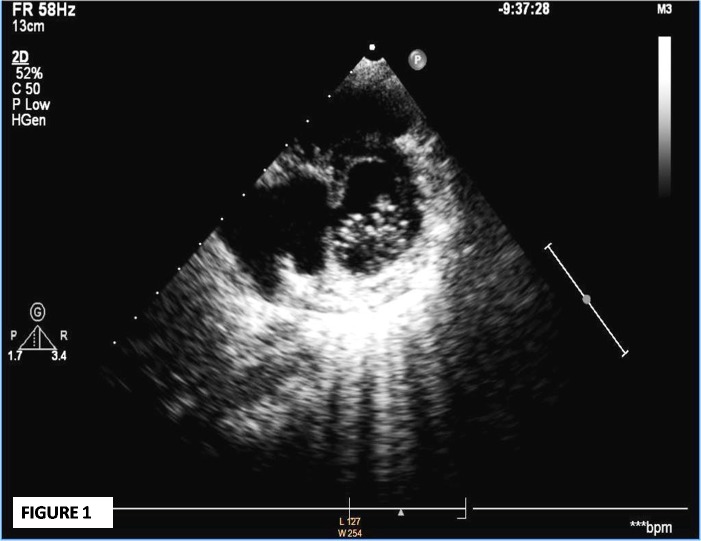
Figure 1:Parasternal short axis view at mitral valve level demonstrating a large aneurysmal sac on left of image with echo dropout at inferior septum.

**Figure:**
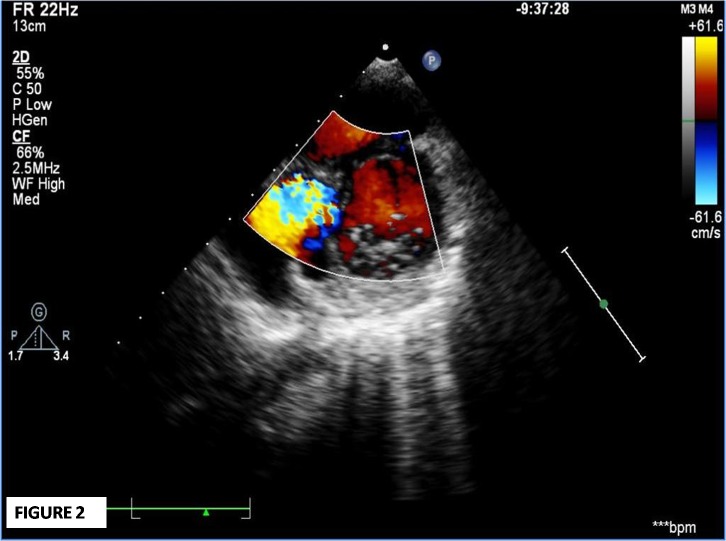
Figure 2:Parasternal short axis view at mitral valve level demonstrates communication between aneurysmal sac and left ventricular cavity on color flow.

**Figure:**
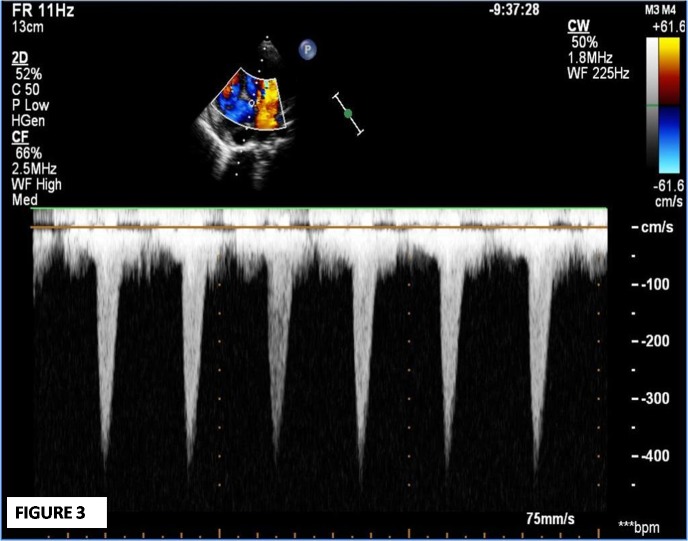
Figure 3:Continuous wave (CW) doppler interrogates the flow from left ventricular cavity toaneurysmal cavity during systole.

## DISCUSSION

Formation of ventricular pseudo-aneurysm may be due to the inflammation resulting in a small abscess within the myocardium that gradually perforate the ventricular wall and contained within the thickened pericardium or fibrous tissue.[1] There are several theories proposed in previous reports regarding the pathogenesis that include production of staphylokinase enzyme (converts plasminogen to plasmin, which breaks down the collagen).[6,8] These mechanisms have been suggested because in almost all the cases, there is a definite gap after the patient gets symptomatically better with intravenous antibiotics. In almost all the reported paediatric cases, the condition usually follows disseminated staphylococcal infection or septicaemia.[1-8] Either endocarditis or myocarditis initiating the process of formation of pseudo-aneurysm is unlikely in present case.

In children, staphylococcus aureus is the commonest organism causing infective pericarditis. The most common source of disseminated staphylococcal septiacemia is usually an abscess. In the index case the primary source of infection was successfully drained. The pericardial effusion, which probably was infected in this case, might spread the infection to the myocardium as reported by others.[1-8] Pseudo-aneurysm following staphylococcal septicaemia is rare a high index of suspicion and timely intervention can result in favourable outcome as happened in the index case.

## Footnotes

**Source of Support:** Nil

**Conflict of Interest:** None declared

